# Notes

**Published:** 1898-03-05

**Authors:** 


### NOTES.

Mr. Thomas Naish has generously promised to provide at his own expense an isolation hospital for Gosport, capable of accommodating 16 patients.

An admirable new infirmary has just been added to the Newton Abbot Workhouse, including three lying-in wards and accommodation for some 120 sick and infirm inmates.

Mr. Arthur Cross has announced his intention of building and endowing a convalescent home, entirely for the use of the poor of St. Michael's parish, Chester Square.

The Clothworkers' Company has sent a subscription of £200 to the London Hospital Maintenance Fund, and has promised to subscribe £200 annually for the next five years. The hospital has just issued its fifth quinquennial appeal for funds to carry on its work for the next five years. An anonymous sum of £2,000 has just been sent to the hospital through Messrs. Glyn, Mills, Currie, and Co.

At the last quarterly Court of Governors of the Devon and Exeter Hospital new statutes were passed which admit a proportion of the medical and surgical staff to the governing body. Dr. Davy said: "There was a wide distrust among the general public as to the management of the hospital at present, which accounted for the deficit in funds. It was entirely owing to the medical staff that the old nursing scheme—which was a disgrace to the hospital—had been reconstructed, and it was right and proper for the medical staff to have a voice in the government of all hospitals."

